# Development of a Stable MGAT1^−^ CHO Cell Line to Produce Clade C gp120 With Improved Binding to Broadly Neutralizing Antibodies

**DOI:** 10.3389/fimmu.2018.02313

**Published:** 2018-10-05

**Authors:** Rachel C. Doran, Bin Yu, Meredith Wright, Sara M. O'Rourke, Lu Yin, Jennie M. Richardson, Gabriel Byrne, Kathryn A. Mesa, Phillip W. Berman

**Affiliations:** ^1^Department of Biomolecular Engineering, University of California, Santa Cruz, Santa Cruz, CA, United States; ^2^Department of Molecular, Cell and Developmental Biology, University of California, Santa Cruz, Santa Cruz, CA, United States

**Keywords:** HIV, gp120, Clade C, vaccine, glycosylation, cell line

## Abstract

The high rate of new HIV infections, particularly in Sub-Saharan Africa, emphasizes the need for a safe and effective vaccine to prevent acquired immunodeficiency syndrome (AIDS). To date, the only HIV vaccine trial that has exhibited protective efficacy in humans was the RV144 study completed in Thailand. The finding that protection correlated with antibodies to gp120 suggested that increasing the quality or magnitude of the antibody response that recognize gp120 might improve the modest yet significant protection (31.2%) achieved with this immunization regimen. However, the large-scale production of rgp120 suitable for clinical trials has been challenging due, in part, to low productivity and difficulties in purification. Moreover, the antigens that are currently available were produced largely by the same technology used in the early 1990s and fail to incorporate unique carbohydrates presented on HIV virions required for the binding of several major families of broadly neutralizing antibodies (bNAbs). Here we describe the development of a high-yielding CHO cell line expressing rgp120 from a clade C isolate (TZ97008), representative of the predominant circulating HIV subtype in Southern Africa and Southeast Asia. This cell line, produced using robotic selection, expresses high levels (1.2 g/L) of the TZ97008 rgp120 antigen that incorporates oligomannose glycans required for binding to multiple glycan dependent bNAbs. The resulting rgp120 displays a lower degree of net charge and glycoform heterogeneity as compared to rgp120s produced in normal CHO cells. This homogeneity in net charge facilitates purification by filtration and ion exchange chromatography methods, eliminating the need for expensive custom-made lectin, or immunoaffinity columns. The results described herein document the availability of a novel cell line for the large-scale production of clade C gp120 for clinical trials. Finally, the strategy used to produce a TZ97008 gp120 in the MGAT^−^ CHO cell line can be applied to the production of other candidate HIV vaccines.

## Introduction

While the availability of anti-retroviral drug prevention and treatment strategies has significantly reduced mortality associated with HIV infection, the endurance of HIV transmission remains a major public health concern. This is particularly true for Sub-Saharan Africa and South Asia, where the majority of new infections are predicted to occur over the next decade (1). Thus, an effective vaccine remains a relevant strategy to stop the spread of HIV. The RV144 HIV vaccine trial completed in Thailand (2003-2009) provided evidence that a prime-boost vaccine concept could provide modest protection (31%, *p* = 0.04) from HIV infection (2, 3). The RV144 protocol employed a recombinant canarypox virus vector (VCP1521) to stimulate a cell-mediated immune response, with bivalent recombinant gp120 (rgp120) immunogens (AIDSVAX B/E), to promote an anti-gp120 antibody response (3). Follow-up studies correlating protection in RV144 with non-neutralizing antibodies against gp120, but not cell-mediated immunity, supported a role for the rgp120 immunogen in the observed protection (2). Following the RV144 trial, multiple families of broadly neutralizing antibodies (bNAbs) that bind oligomannose structures were identified, highlighting the importance of specific glycoforms (mannose-5 and mannose-9) on the HIV envelope glycoprotein (Env) (4–8). However, the rgp120 immunogens used in the RV144 trial were expressed in CHO cells, and therefore enriched for complex, sialic acid containing N-linked glycans that preclude binding glycan dependent bNAbs (9). Together, these observations provided justification for investigation of gp120-based immunogens incorporating the oligomannose (mannose-5 and mannose-8/9) glycoforms found on native virions and targeted by bNAbs (8, 10, 11).

We screened a diverse panel of clade C gp120 protein isolates expressed in HEK 293 cells to identify a clade C envelope protein that displayed above average binding to different bNAbs. To express the clade C rgp120, we employed a novel cell line (MGAT1^−^CHO), created in our laboratory through the use of the CRISPR/Cas9 gene editing to inactivate the Mannosyl (Alpha-1,3-)-Glycoprotein Beta-1,2-N-Acetylglucosaminyltransferase (MGAT1) gene (12). The resulting cell line expresses rgp120 proteins containing N-linked mannose-5 or earlier intermediate glycoforms that are recognized by various families of glycan dependent bNAbs. This strategy is advantageous to previous approaches to manipulate glycosylation on rgp120 (i.e., expression in HEK 293 GNTI^−^ cells, or with the use of glycosidase inhibitors such as kifunensine) in that it can be used as part of a biopharmaceutical production system amenable to current Good Manufacturing Practices (cGMP). Additionally, expression of rgp120 in the MGAT1–CHO cell expression system reduces heterogeneity in net charge as compared to CHO-expressed rgp120. Such homogeneity of MGAT1–CHO derived rgp120s facilitated the development of an ion-exchange based purification method that obviated the need for custom affinity-chromatography resins previously used for purification of rgp120 immunogens (13). Here we compare the properties of a clade C rgp120, TZ97008, produced in normal CHO cells, resembling those used to produce gp120 for previous (3, 14, 15) and current clinical trials (16), with TZ97008-rgp120 produced in the MGAT1–CHO cell line. Our results demonstrate that the MGAT1–CHO expression system provides a cost-effective approach for the production of the clade C TZ97008 rgp120 displaying oligomannose glycoforms that both simplifies down-stream purification and improves the binding of bNAbs.

## Materials and methods

### Clade C gp120 screening

The panel of clade C gp120s was assayed for bNAb binding by Fluoresence ImmunoAssay (FIA). Antigen was diluted to 2 μg/mL in PBS and coated onto 96 well black-microtiter plates (Greiner, Bio-One, USA) at 4°C overnight. Plates were blocked in PBS with 1% BSA for 2 h. Three-fold dilutions of antibody were added, followed by a 1:3,000 dilution of Alexa Fluor 488 conjugated goat-anti-human polyclonal secondary (Jackson ImmunoResearch Laboratories, West Grove, PA). Incubations were performed for 90 min (23°C) in blocking buffer and preceded by a 4x wash in PBST unless otherwise noted. The panel of 10 clade C, gD tagged envelope proteins was expressed in HEK 293 cells as described previously (17). Recombinant gp140 from the 1086 strain of HIV-1, contributed by Drs. Barton F. Haynes and Hua-Xin Liao, was obtained from NIH AIDS Reagent Program, Division of AIDS, NIAID, NIH (18, 19). The TV1 gp120 expressed in 293 HEK cells was obtained from Immune Tech Corporation (New York, NY). The PG9, PGT121, PGT128, and VRC01 bNAbs were produced in our laboratory in 293 HEK cells based on published sequences. The 10-1074 bNAb was obtained through the NIH AIDS Reagent Program, Division of AIDS, NIAID. Affinity purified polyclonal goat antibodies, raised against an equimolar mixture of purified gp120s from clade B (MN), clade C (CN97001), and clade E (TH023) gp120s, was used as a positive control for the FIA binding assays.

Area under the curve (AUC) calculations were performed using GraphPad Prism version 6.0 for Mac, GraphPad Software, La Jolla California USA. To obtain a relative ranking of overall bNAb antigenicity, individual area under the curve (AUC) analyses were taken of individual gp120 binding curves to each of the 5 bNAbs, and the sum of these AUC values for each gp120 antigen was ranked. Rank scores from 1-12 were awarded to the 12 envelope antigens based on calculated AUC values, with a rank score of one awarded to the envelope protein exhibiting the poorest binding (lowest calculated AUC) to a bNAb, to a rank score of 12 awarded to envelope antigen exhibiting the highest binding (highest calculated AUC). The AUC rank value scores were summed, and the subsequent values were used to determine relative gp120 antigenicity and to select a gp120 sequence for further development. The clade C consensus nucleic acid sequence was obtained Los Alamos National Laboratory 2010 HIV1 Env Reference Alignment and was translated using Geneious 10.2.3 (20). Sequences were globally aligned using Geneious alignment algorithm with free end gaps (Cost matrix = Blosum62, Gap open penalty = 12, Gap extension penalty = 3, Refine iterations = 2). The consensus sequence was trimmed to match the known amino acids in TZ97008 (V42–A525, HXB2 reference numbering).

### Development of a MGAT1^−^ CHO cell line to express TZ97008-rgp120

Suspension adapted CHO-S cells were obtained from Thermo Fisher (Thermo Fisher, Life Technologies, Carlsbad, CA). The TZ97008 MGAT1^−^ CHO cell line was developed following a protocol described previously in O'Rourke et al. (21). Cells were transfected with a modified pCDNA3.1 expression vector (22) encoding TZ97008 and the gene encoding resistance to Geneticin. The gp120 transcription unit included an N-terminal purification tag from Herpes Simplex Virus glycoprotein D (gD) as described previously (13). The cells were transfected using an STX electroporation device (MaxCyte Inc., Gaithersburg, MD) according to manufacturer's protocols. Following transfection, cells were serially diluted from 500-5,000 cells/mL in semi-solid CHO-Growth A media with 1XHT and L-glutamine (Molecular Devices, Sunnyvale, CA), 500 μg/ml G418, and 10 μg/ml Alexa Fluor 488 conjugated goat anti-gp120 polyclonal antibodies reactive with clade B, AE, and C rgp120s as described previously (21). Colonies expressing rgp120 were selected on basis of fluorescence and default selection parameters using the ClonePix2 (Molecular Devices, Sunnyvale, CA).

Cell culture in shake flasks was performed in BalanCD® CHO Growth A medium (Irvine Scientific, Santa Ana, CA) supplemented with 1XHT (Corning, Corning NY), 10% Proyield® Cotton seed protein (DOMO, Netherlands), and 0.25 mM glutaMAX (Sigma-Aldrich Chemicals, St. Louis, MO). Cell viability was assessed via with trypan blue exclusion and cell counts and viability were monitored using the TC20TM automated cell counter (BioRad, Hercules, CA). The concentration of rgp120 in growth conditioned cell culture supernatant was quantitated using a capture ELISA. Briefly, rgp120 in cell culture supernatant was captured on 96 well Maxisorp plates via a mouse monoclonal antibody (34.1) to an N-terminal purification tag (23), incubated with a 2 μg/mL dilution of purified goat-anti-rgp120 polyclonal antibody, and detected with a 1:3,000 dilution of bovine-anti-goat HRP conjugated polyclonal and OPD substrate. Reactions were stopped with 3M sulfuric acid. Dilutions were performed in blocking buffer (1% BSA in PBS) and all assays were performed in duplicate. For comparison, CHO derived TZ97008-rgp120 was expressed via transient transfection using a method as previously described (24).

### Purification of TZ97008 rgp120 proteins

Supernatants containing MGAT1^−^ CHO expressed TZ97008 rgp120 were concentrated and buffer exchanged to 10 mM TRIS, pH = 8.0 buffer (Vivaflow 200 Sartorius, Göttingen, Germany). Eluent was monitored at the 280 nm wavelength using an Akta purifier (GE healthcare Chicago, IL). Concentrated and buffer-exchanged MGAT1^−^ CHO supernatant containing TZ97008 rgp120 was passed through a 5 cm anion exchange (HiTrap Q FF column, GE Healthcare, Chicago, IL) and flow through fractions containing gp120 were collected. These fractions were further purified via size exclusion chromatography using a 60 cm Highload Superdex 200pg column (GE Healthcare) in pH 8 TBS (Tris-buffered Saline) buffer. CHO-S expressed TZ97008 rgp120 was purified by affinity chromatography against the N-terminal gD tag, followed by size exclusion chromatography, as previously described (9).

### Protein concentration and recovery calculations

Purified rgp120 from the relevant cell expression system was used as a standard to determine rgp120 concentration in in cell culture supernatants, column flow-through, and eluates associated with each individual purification step. Protein was quantified using a concentration dependent analysis protocol with the Biacore X100+ software V2.0.1 (GE Health Sciences). The conformation dependent but glycan independent bNAb VRC01 was coupled to the chip via an anti-human Fc polyclonal and used as a capture mAb for concentration analysis. The concentration of either anion exchange- or immune-affinity chromatography purified TZ97008 rgp120 from MGAT1^−^ CHO cell line was confirmed by BCA (Thermo Fisher, Waltham, MA).

### Physical and antigenic analysis of purified rgp120

#### SDS-PAGE, isoelectric focusing (IEF), and endoglycosidase digestions

SDS-PAGE of purified rgp120 proteins was performed as previously described (25). Purified protein samples were run on a Bis-Tris 4–12% gradient gel (Invitrogen, Carlsbad, CA) and stained with SimplyBlue SafeStain (Invitrogen, Carlsbad, CA). Endoglycosidase H (Endo H) and PNGase F digest kits were purchased from New England Biolabs (Ipswich, Massachusetts). Briefly, 200 μg of rgp120 was reduced with provided denaturation buffer and boiled for 10 min at 100°C. Denatured rgp120 was then mixed with reaction buffer and 5,000 units of enzyme and digests were incubated for 24 h at 37°C. TZ97008-rgp120 from CHO and MGAT1^−^ CHO expression systems, purified by affinity or ion exchange purification methods, respectively, were analyzed by two-dimensional isoelectric focusing gel analysis as previously described (9). Proteins were run on ReadyStrip^TM^ IPG strips (11 cm, pH 3-10, Bio-Rad, Hercules, CA), and resolved using a Protean® IEF Cell (Bio-Rad, Hercules, CA) to establish the horizontal dimension based on the protein isoelectric focusing point. The vertical dimension, determined by protein molecular weight and shape, was established by running strips along 4–15% polyacrylamide tris-glycine gels (Bio-Rad, Hercules, CA). Proteins were stained with SimplyBlue SafeStain (Invitrogen, Carlsbad, CA). Amyloglucosidase from *Aspergillus niger* (97 kDa, pI = 3.6) and/or carbonic anhydrase isozyme II (29kDa, pI = 5.9) from bovine erythrocytes (Sigma-Aldrich Chemicals, St. Louis, MO) were included as internal pI standards.

#### Reverse phase high performance liquid chromatography (RP-HPLC)

RP-HPLC was performed using a Shimazu 10A VP serial HPLC system (Columbia, Maryland), and run on a 150 mm^*^2.1 mm Betabasic C18 column (Thermo Scientific, Waltham, MA). Solutions were run at a 1% per minute (5% acetonitrile to 60% acetonitrile in water) gradient, and eluent was monitored at UV 214 nm.

#### Antibody binding assays

Protein-antibody binding kinetics were assessed via surface plasmon resonance (SPR) using the Biacore X100+ (GE Healthcare, Chicago, IL). Standard amine coupling methods were used to couple anti-human Fc to a CM5 sensor chip. Assayed bNAbs were injected until 100-150 RU signal increase was achieved. Following, serial dilutions from 0.25 to 80 nM of TZ97008-rgp120 in HBS-EP buffer were injected, and ligand capture protocols (three min at 30 μL/min followed by 10 min dissociation) were used to determine kinetics. Channels were regenerated with 3M MgCl_2_. Biacore evaluation software was used to subtract blank injection references and determine K_d_ measurements (V 2.0.1, GE Health Sciences). Fluorescence immunoassays were performed as capture fluorescence immunoassay as described previously (24).

## Results

### Screening clade C HIV envelope proteins

**Figure 1:**
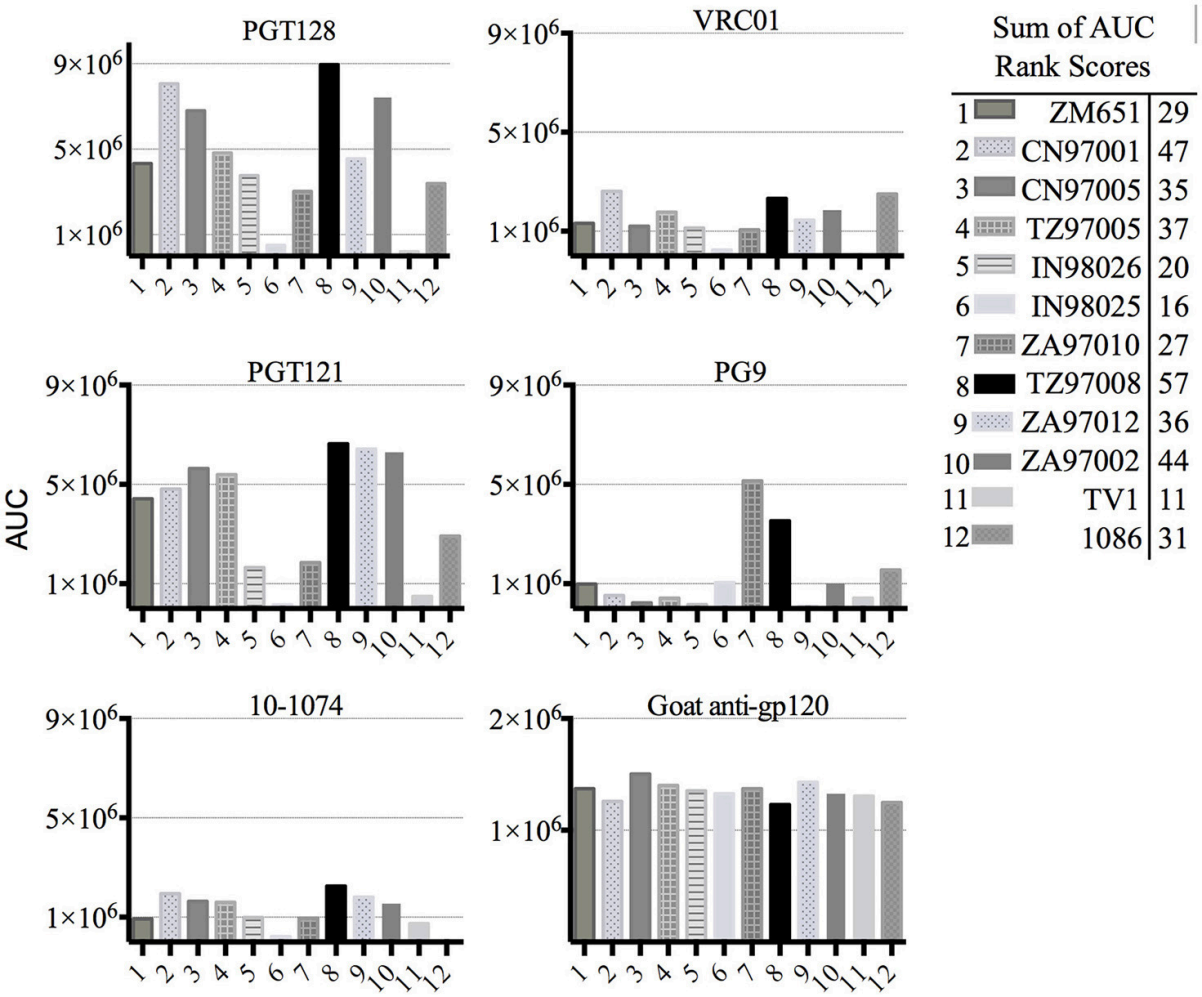
Screen of bN-mAb binding to clade C rgp120 proteins of diverse geographic origins. Clade C gp120 proteins expressed in 293 HEK cells were compared by FIA for binding to a panel of five prototypic bNAbs. All rgp120 proteins except the TV1 and 1,086 are appended with an N-terminal gD purification tag as described previously (17). To obtain a relative ranking of overall bNAb antigenicity, individual Area under the Curve (AUC) analyses were calculated for individual envelope protein binding curves as described Materials and Methods. The AUC rank scores for all of the 5 bNAbs assayed were summed, and the cumulative AUC score of each envelope protein antigen is shown in in the included table.

**Figure 2:**
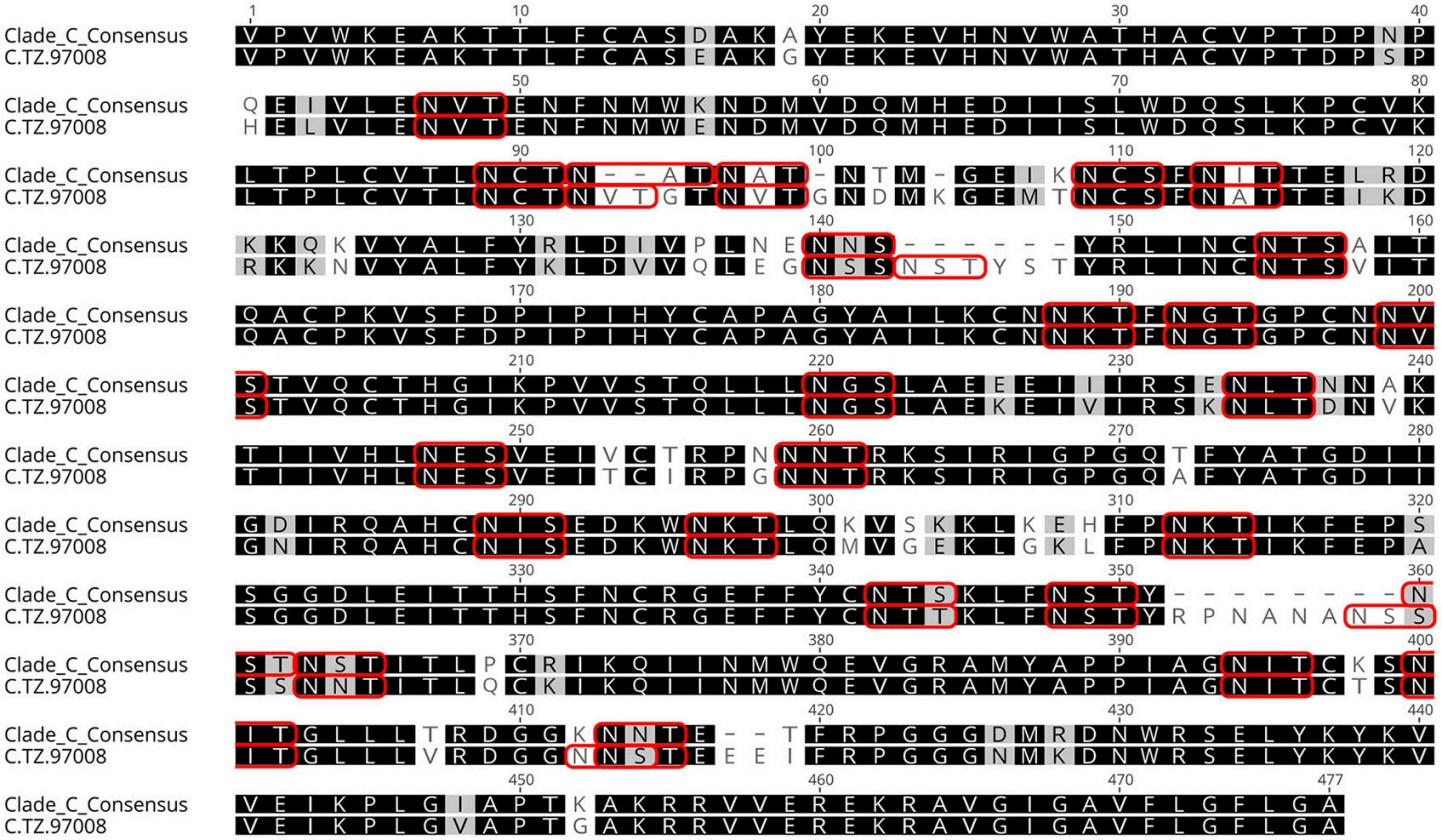
Tz97008 alignment to Clade C consensus. TZ97008 and LANL clade C consensus amino acid sequences were aligned in Geneious 10.2.3 (1) to compare TZ97008 to the most common features in clade C. Potential N-linked glycosylation sites are emphasized within the alignment with a box.

Previous studies have indicated that binding of gp120s to different bNAbs varies considerably as a result of primary sequence, glycan occupancy, and higher order structure (26, 27). Therefore, we assembled a panel of clade C envelope proteins to identify a clade C gp120 able to bind multiple families of bNAbs. The panel included rgp120s of diverse geographical origins such as Sub-Saharan Africa, China and India (17), as well as the TV1-rgp120 and 1086-rgp140 sequences similar to those currently being tested in RV144 follow-up studies in South Africa (HVTN702) (28). A fluorescence immunoassay (FIA) was used to compare gp120 binding to four diverse and genetically distinct families of bNAbs (PG9, PGT128, PGT121, and VRCO1). A goat polyclonal antibody with reactivity against clade C strains was included as a positive control. Area under the curve (AUC) value scores were derived from FIA binding curves and used to rank the magnitude and breadth of bNAb binding for the panel of envelope proteins (see Materials and Methods). The AUC rank value scores were summed. From these sums, the TZ97008-rgp120 was identified to possess the highest overall breadth and magnitude of binding to assayed bNAbs (Figure 1). The TZ97008 rgp120 exhibited the best binding to the PGT128 bNAb that is binds a glycan epitope in the stem of the V3 domain (29). Additionally, it was amongst the top quartile of binding to the PG9 bNAb that binds a glycan dependent epitope in the V1/V2 domain (4), the CD4-binding site binding bNAb VRC01 (30), and the V3 glycan dependent bNAb PGT121 (31). TZ97008 and LANL clade C amino acid sequences were aligned in Geneious 10.2.3 (1) to compare TZ97008 to the most common features in clade C (Figure 2). We found that TZ97008 has 84.1% pairwise identity to the LANL clade C consensus amino acid sequence. Notably, and nearly all the potential N-linked glycosylation sites (PNGS) are aligned. Overall, TZ97008 appeared highly similar to the clade C consensus sequence with no unusual features deviant from the clade C consensus.

### Development of the 3E5 cell line to express TZ97008 rgp120

**Figure 3:**
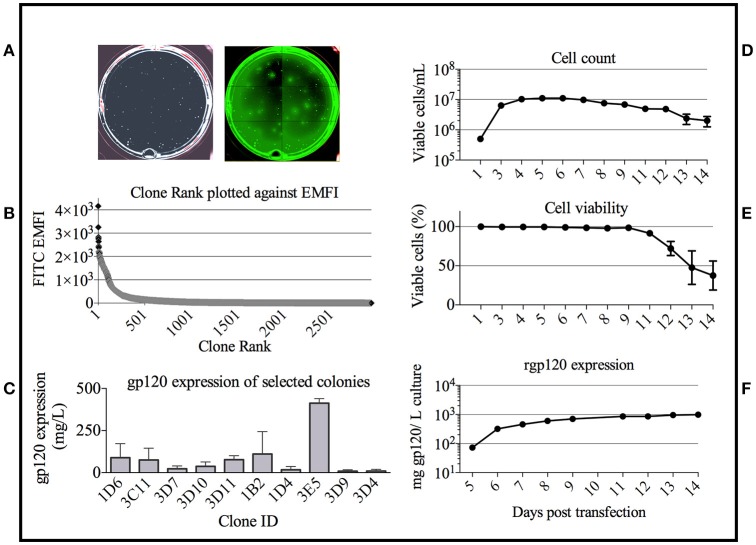
Colony selection of MGAT1 transfected cells. **(A)** 10,000 colonies of cells transfected with a plasmid encoding TZ97008-rgp120 were analyzed for rgp120 expression using the ClonePix 2 robot. The number of colonies was visualized under white light, and the relative magnitude of gp120 expression was determined by size and intensity of halos visualized under fluorescent light (470nm excitation and 535nm emission wavelength filters). **(B)** Colonies were ranked by exterior mean fluorescent intensity (EMFI) as readout of rgp120 expression and EMFI was plotted as a function of clone rank. **(C)** Following growth and selection, the 10 best growing clones were grown in six-day batch culture and assayed for gp120 expression by quantitative ELISA. Binding curves were compared to a standard curve of known protein concentration. From the expression screen, the highest expressing clone (3E5) was expanded for an 11-day fed-batch fermentation run. Cells were monitored for; **(D)** cell count, **(E)** viability, and **(F)** rgp120 expression. Assays were performed in duplicate and error bars indicate Standard Deviation (SD).

**Figure 4:**
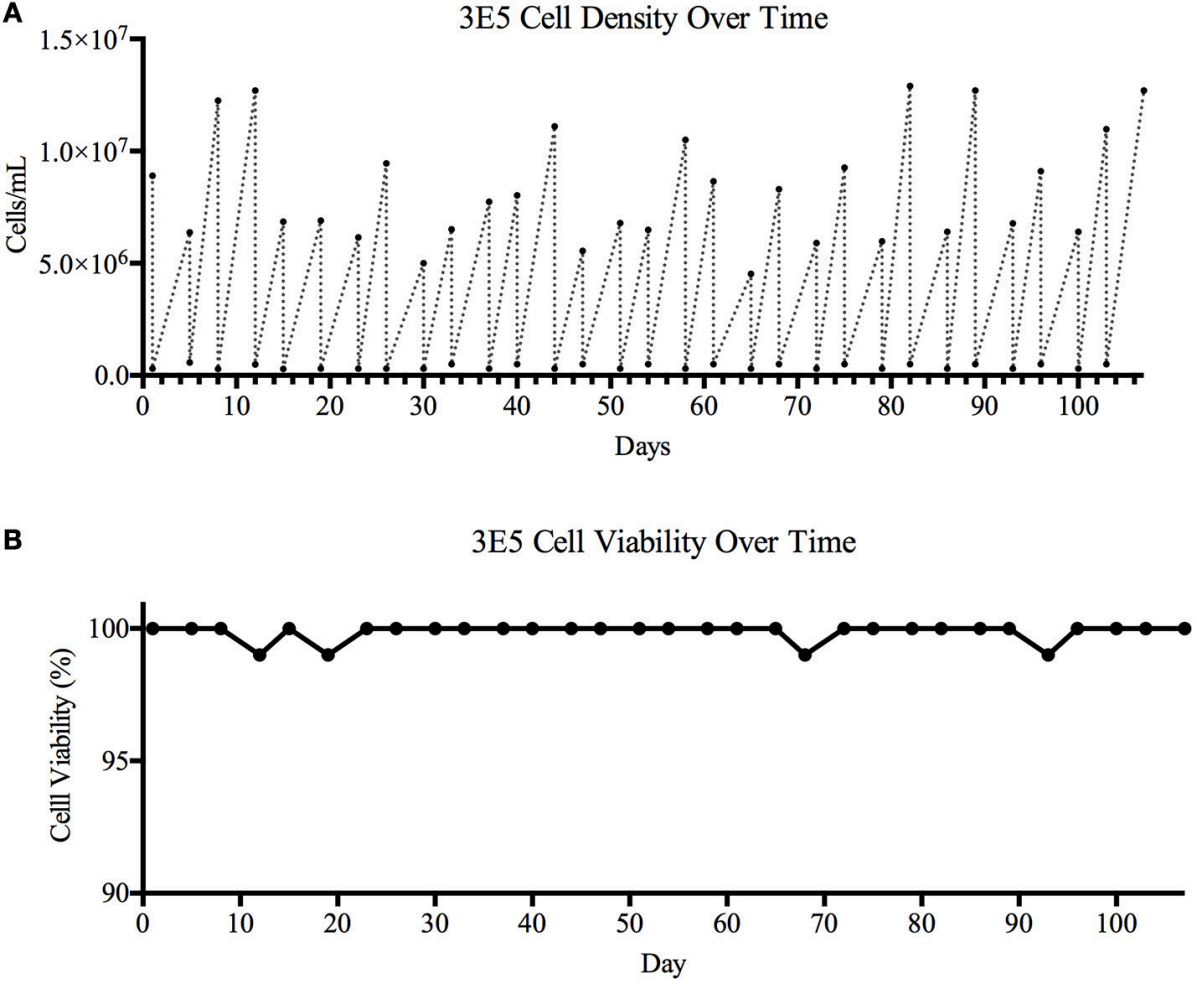
Cell viability over time. A 106-day study designed to determine cell line stability and viability over time was carried out. The TZ97008 expressing 3E5 MGAT1^−^ CHO cell line was entered into a viability stability assay and carried for a total of 31 passages. The 3E5 cell line culture was adapted to suspension culture with a fractioned (300μg/mL) concentration G418 in BalanCD® CHO Growth A medium and incubated at 37°C. **(A)** Cell density and **(B)** cell viability was measured using Trypan Blue staining before cultures were diluted down to ~3E5 cell/mL. Following dilution, cells were allowed to grow for 3–4 days before cell counts and viability were measured and cultures were again diluted.

Based on the bNAb binding results, we pursued development of a stable cell line expressing TZ97008-rgp120. To this end, we utilized a cell-line development method developed by O'Rourke et al. (21). The MGAT1^−^ CHO cell line was used as a parental cell line, as it possesses a mutation in the gene encoding Mannosyl (Alpha-1,3-)-Glycoprotein Beta-1,2-N-Acetylglucosaminyltransferase (Mgat1) and therefore incorporates predominantly mannose-5 or earlier (i.e., mannose-8/9) glycoforms (12). Such oligomannose glycoforms have been found to contribute to gp120 recognition by different families of bNAbs (4, 32). MGAT1^−^ CHO cells were transfected via electroporation with a pCDNA3.1 expression vector containing the TZ97008-rgp120 gene. A total of 1.5 × 10^5^ transfected cells was serially diluted and grown on semi-solid media containing G418 and anti-gp120 polyclonal antibodies labeled with Alexa Fluor 488 (see Materials and Methods). After 14 days, white light and fluorescent images were acquired using the ClonePix2 (Figure 3A). Acquired images were used to screen the ~7,000 colonies and identify colonies actively secreting rgp120. The size and intensity of the fluorescent halo resulting from immunoprecipitation by Alexa Fluor 488 conjugated anti-gp120 polyclonal antibody around rgp120 secreting cells was measured as exterior mean fluorescence intensity (EMFI), and used in conjunction with additional default ClonePix2 selection parameters to select colonies for further development. Figure 3B depicts the relative EMFI of imaged colonies. A total of 384 colonies were selected and expanded into 96 well cultures. As colonies were passaged, cell growth, as determined by well confluence at time of passage, and rgp120 expression, as determined by ELISA, were monitored. Following four passages, the 10 clones exhibiting consistent growth and gp120 expression were quantified for rgp120 expression to select for the highest producing cell line, designated 3E5 (Figure 3C). From the expression screen, the highest expressing clone (3E5) was expanded to a 125 mL shake flask and monitored for cell count and viability over a 14-day fermentation process (Figures 3D,E). Growth conditioned cell culture medium was analyzed by ELISA for each clone and expression levels as high as 1,200 mg/L expression were obtained from fed batch cultures of the TZ97008 MGAT1^−^ CHO 3E5 line (Figure 3F). The 3E5 clone was additionally entered into a 63-day stability assay, over which cells were passaged for an additional 30 passages (Figure 4A) and observed to maintain >98% viability (Figure 4B).

### Development of a conventional purification process for TZ97008 rgp120

A high degree of net charge heterogeneity present on envelope proteins produced in CHO cell previously necessitated gp120 purification schemes to employ immunoaffinity or lectin affinity chromatography (13, 16, 33–36). To eliminate complications introduced by such methods and to efficiently process the large amounts of protein, we developed a purification process that does not rely on lectin or immunoaffinity based resins. While the negatively charged sialic-acid incorporated into gp120 produced in CHO cell lines results in a product displaying a broad spectrum of isoelectric points (pI 3.5-7.5) (9), rgp120 expressed in cell lines incorporating exclusively oligomannose N-linked glycans and exhibit pIs of a smaller range (9). The lower degree of net charge heterogeneity on TZ97008-rgp120s produced in the 3E5 cell line permitted the use of ion exchange and size exclusion chromatography. The use of ion exchange and size exclusion chromatography additionally offers a cost-effective purification alternative that is amenable to optimization for high-throughput systems. For this method, growth conditioned cell culture medium from the 3E5 cell line was buffer exchanged to pH = 8.0 in a low salt solution and passed through Hitrap Q FF anion exchange column. Using this strategy, CHO host cell proteins were mostly retained on the column, while rgp120 was present in the flow through fractions. The flow through containing rgp120 was then passed over a size exclusion column for final purification.

**Figure 5:**
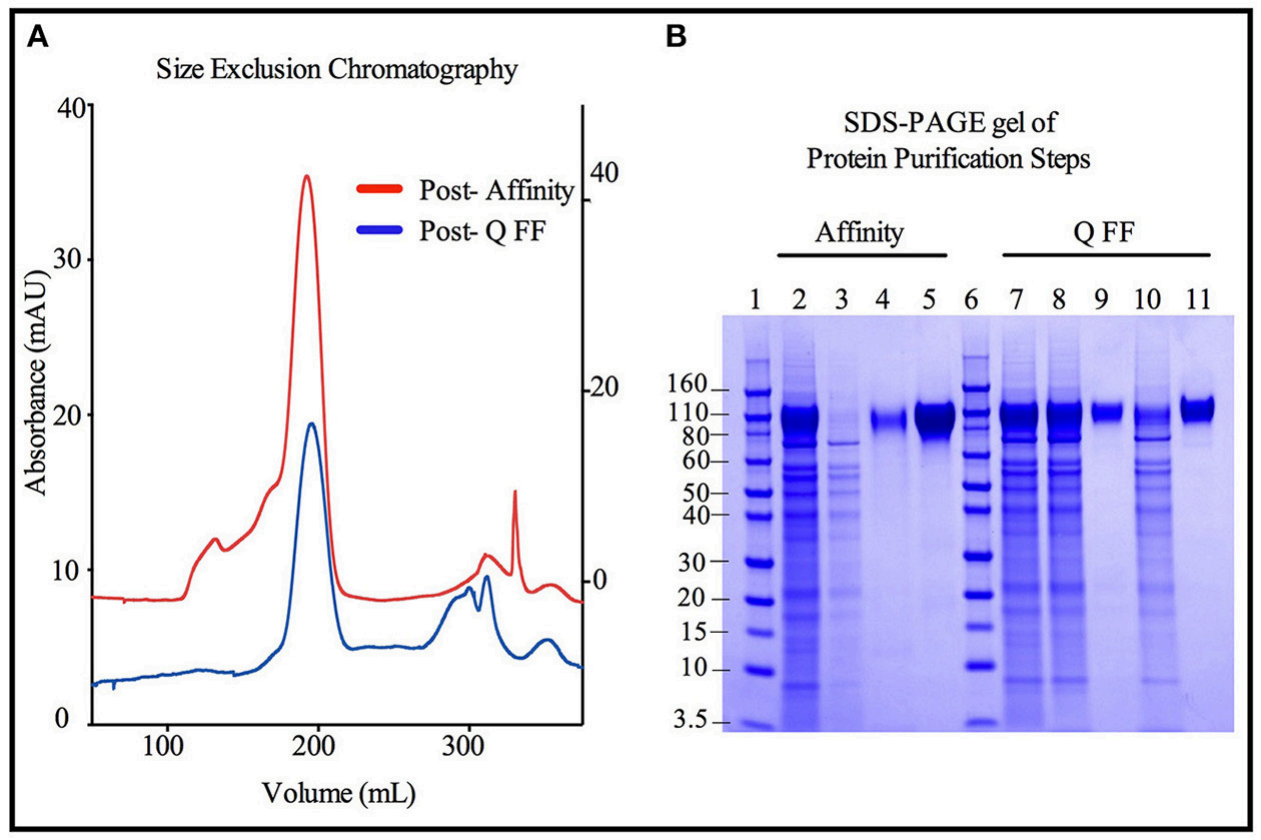
Comparison of immunoaffinity and ion-exchange chromatography purification strategies for 3E5 MGAT1^−^ CHO expressed TZ97008-rgp120. Two methods were compared for the purification of 3E5 MGAT1^−^ CHO expressed TZ97008-rgp120. The first method reconstructed the method used to purify the rgp120 immunogens used in the RV144 clinical trial (3) and incorporated an affinity chromatography column that purified gp120 via an N-terminal gD tag as previously described (13). Briefly, gp120 molecules retained on the affinity chromatography column were selectively eluted at low pH. Following neutralization and buffer exchange. The second method incorporated anion exchange chromatography (QFF) as described in Materials and Methods. Briefly, growth conditioned cell culture medium was concentrated and buffer exchanged. The resulting solution was passed through a HiTrap QFF column, wherein gp120 molecules flowed through the column with a large proportion of CHO proteins were retained on the column. Size exclusion chromatography (SEC) was used to further purify protein products from Affinity and QFF methods **(A)**. A chromatograph depicts the SEC elution profiles of the CHO or 3E5 derived TZ97008-rgp120 proteins initially purified by the Affinity method (red) and the QFF method, respectively (blue). The left Y-axis corresponds to the Post-Q FF chromatograph, and the right Y-axis corresponds to the Post- Affinity chromatograph. **(B)** The products resulting from the Affinity (lanes 2–5) and QFF (lanes 7–11) purification methods were compared by SDS-PAGE. Samples from each step of the purification were treated with SDS-PAGE sample buffer and loaded onto 4–12% Bis-tris gels. Starting cell culture supernatants from the 3E5 MGAT1^−^ CHO fermentation cultures are shown (lanes 2 and 7). Flow through and eluate fractions of CHO culture supernatants after passage through immunoaffinity column (lanes 3 and 4, respectively) and the main rgp120 peak from the SEC column (lane 5) are shown. Flow through fractions of 3E5 MGAT1^−^ CHO cultures following buffer exchange and the QFF column flow throughs are shown in lanes 8 and 9, respectively. Lane 10 and 11 indicate the proteins retained on the QFF column that eluted with salt solutions (predominantly CHO host cell proteins), and the major rgp120 containing fraction of SEC, respectively. Molecular weight standards are included (lanes 1 and 6).

Size exclusion chromatographs of MGAT1^−^ CHO expressed TZ97008-rgp12 purified by either the affinity or anion exchange methods are compared in Figure 5A. Supernatant, flow through and elution of affinity column and anion exchange column, and final TZ97008 rgp120 products from both methods were run on SDS-PAGE in Figure 5B. Together, the chromatographs and the SDS-PAGE gel demonstrate that the TZ97008-rgp120 can be isolated to a clean, homogenous protein by either affinity purification or anion exchange methods. Although some TZ97008-rgp120 bound to the QFF anion exchange column (Figure 5B, lane 10), the majority of the rgp120 could be found in the flow through (Figure 5B, lane 11). The yields of each purification step are summarized in Table 1. In summary, roughly 74% of the original protein was recovered in the flow through of anion exchange chromatography, and 50% final recovery was reached after size exclusion chromatography. From a starting volume of 200 mL of fed-batch 3E5 fermentation supernatant containing an estimated 1.2 mg/mL, 120 mg of MGAT1^−^ CHO TZ97008 rgp120 was recovered for a scaled yield of 600 mg rgp120/L of culture using the anion exchange method. While the anion exchange chromatography method exhibits a comparatively lower recovery than the traditional affinity chromatography approach (74 vs. 93%), it offers distinct, technical advantages. First, this method removes the potentially denaturing low pH elution step used in previous affinity chromatography processes. Additionally, the use of an ion exchange column removes additional cost and documentation associated with production and maintenance of affinity chromatography resins under cGMP standards.

**Table 1:** Comparison of methods for the purification of TZ97008-rgp120.

	**Pre-purification**		**STEP 1 yield**		**STEP 2 yield**	**Final yield**
	**Starting**	**Buffer exchange**	**FT**	**Eluate**	**Eluate**	
Affinity /Size exclusion chromatography	2,650 ug (100%)	N/A	Affinity chromatography		Size exclusion chromatography	70%
			25 ug (1%)	2,460 (93%)	1,848 (75%)	
Q FF/ Size Exclusion chromatography	3,350 ug (100%)	3,350 ug (100%)	Q FF (anion exchange) chromatography		Size Exclusion Chromatography	50%
			2,496 ug (74%)	760 ug (23%)	1,677 (67%)	

*The efficiency of the recovery process for the purification of TZ97008-rgp120 produced in CHO-S cells and MGAT1- CHO cells were compared. TZ97008 produced in CHO-S cells l was purified in two steps; an immunoaffinity chromatography column which captured rgp120 via the N-terminal gD tag (23), followed by size exclusion chromatography (top row). MGAT1^−^ CHO expressed gp120 was purified using an anion exchange chromatography column (QFF), followed by a size exclusion chromatography (bottom row). Surface plasmon resonance (Biacore X100+) was used to determine the concentrations of gp120 before and after each individual purification step. Yields were calculated by dividing protein recovered after each purification step to starting supernatant. Protein not accounted for in the flow through (FT) or eluate calculations was lost during wash steps*.

### Biophysical characterization of purified TZ90008-rgp120

**Figure 6:**
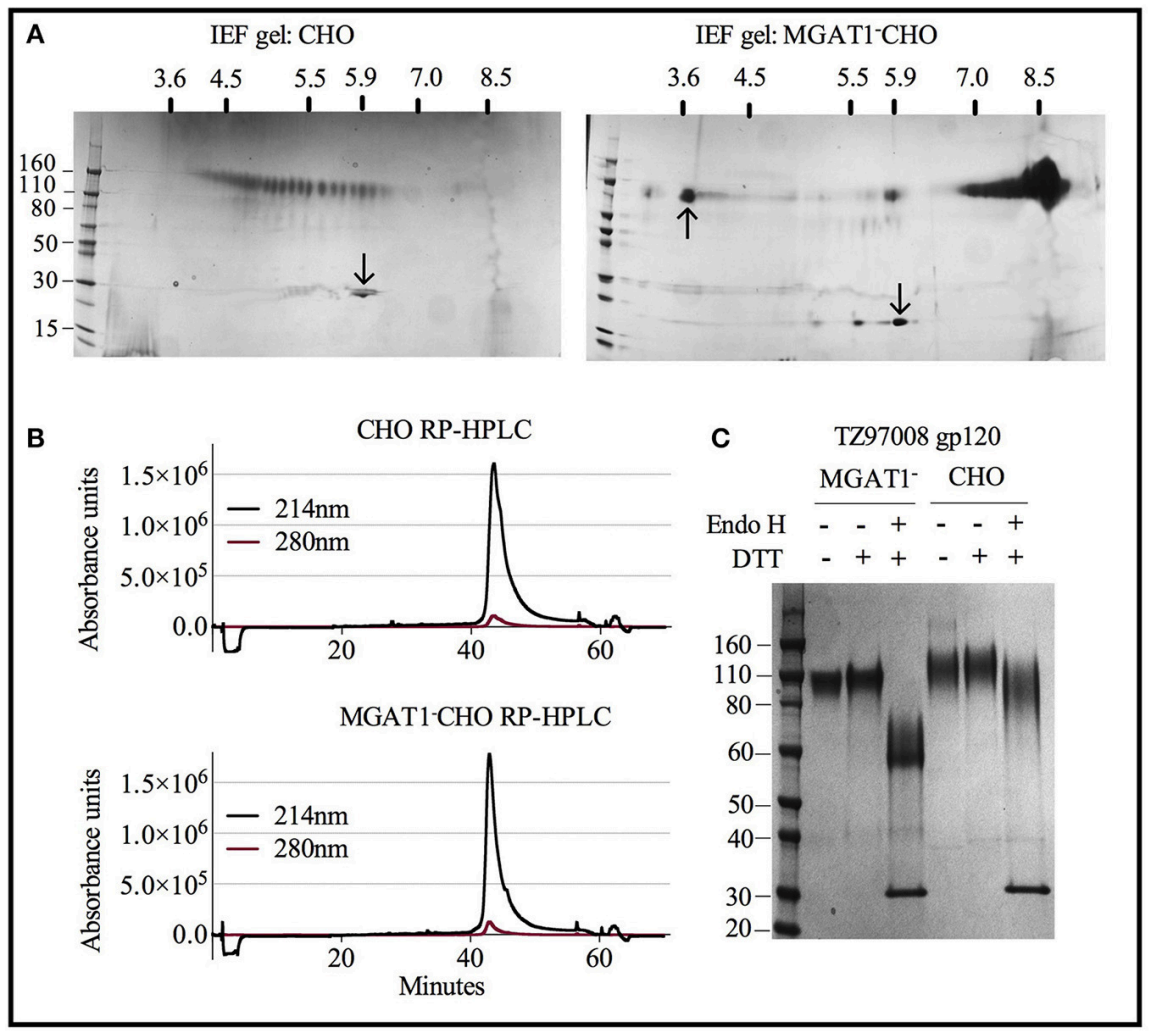
Characterization of purified TZ97008 rgp120 proteins by isoelectric focusing, reverse phase HPLC and endoglycosidase digestion. Purified protein preparations of TZ97008 expressed in CHO-S and MGAT1- CHO and cell lines were analyzed by a variety of methods. **(A)**, 2-dimensional isoelectric focusing gels of TZ97008-rgp120 purified from CHO-S and MGAT1^−^ CHO cells (**A** left and right, respectively). Protein PI standards included the Amyloglucosidase from *Aspergillus niger* (97kDa, pI = 3.6, upward pointing arrow) and/or bovine carbonic anhydrase isozyme II (29kDa, pI = 5.9, downward pointing arrow) are indicated. A protein molecular weight ladder is included on the left-hand side of each panel. **(B)** Purified TZ97008 produced in CHO-S or MGAT1^−^ CHO cells were assayed for purity by RP-HPLC. Absorbances were measured at 214nm (solid line) and 280nm (dashed line) wavelengths **(C)** Purified TZ97008 rgp120 proteins expressed in both cell lines were run on an SDS-PAGE gel to assess for purity, proteolytic degradation (clipping), the presence of dimers, and heterogeneity in N-linked glycosylation. Purified TZ97008 rgp120 proteins expressed by the CHO or MGAT1^−^ CHO 3E5 cell lines were run as reduced and non-reduced samples. Proteins were additionally assayed for Endo H sensitivity to evaluate for presence of simple oligo-mannose terminal glycans, or complex sialic acid containing carbohydrates. For Endo H digestion purified proteins were reduced and subject to Endo H digest overnight at 37°C (**C**, lanes 3 and 6). The Endo H enzyme appears as a 30kDa band present in lanes 3 and 6.

Both CHO and MGAT1^−^ CHO expressed TZ97008-rgp120 preparations could be purified to >95% purity as measured by scanning densitometry of SDS-PAGE gels. However, the differences resulting from cell expression system that facilitated the different purification schemes became apparent upon analysis of purified rgp120 by isoelectric focusing. Whereas, purified rgp120 expressed in the MGAT1^−^ CHO cell line resulted in a bulk protein product of pI ~8.4 (Figure 6A, right), purified TZ97008 rgp120 expressed in the CHO line displayed 25 different gp120 isoforms with PIs spanning pH 3.5 to 6 (Figure 6A, left). When analyzed by RP-HPLC, both CHO and MGAT1^−^ CHO purified preparations ran as a single peak, with the MGAT1^−^ CHO derived protein preparation displaying a slightly more symmetrical peak and smaller trailing shoulder as compared to the CHO derived preparation (Figure 6B). CHO and MGAT expressed proteins both exhibited absorbance peaks starting at 41 min with a minor shoulder eluting at 43 min. However, the CHO expressed gp120 exhibited a wider peak base. Purified rgp120 was assessed for the presence of oligomannose carbohydrate by sensitivity to degradation by the glycosidase Endo H that uniquely degrades oligomannose terminal N-linked glycans. While CHO derived rgp120 remained largely resistant to Endo H digest, the MGAT1^−^ CHO expressed TZ97008 rgp120 was sensitive to Endo H digest, running as a ~60 kDa band post-digest digest (Figure 6C). Additionally, unreduced CHO-S derived TZ97008 rgp120 displayed a faint band at ~200 kDa (Figure 6C), indicative of a small dimerized fraction not present in purified 3E5 MGAT1^−^ CHO derived rgp120 samples. These data indicate that when expressed in a MGAT1^−^ CHO cell line and purified with ion exchange/size exclusion chromatography, TZ97008 rgp120 displayed increased size and glycoform homogeneity and decreased aggregation as compared to when expressed in a normal CHO-S cell line and purified by affinity and size exclusion chromatography.

### bNAb binding to TZ97008-rgp120

**Figure 7:**
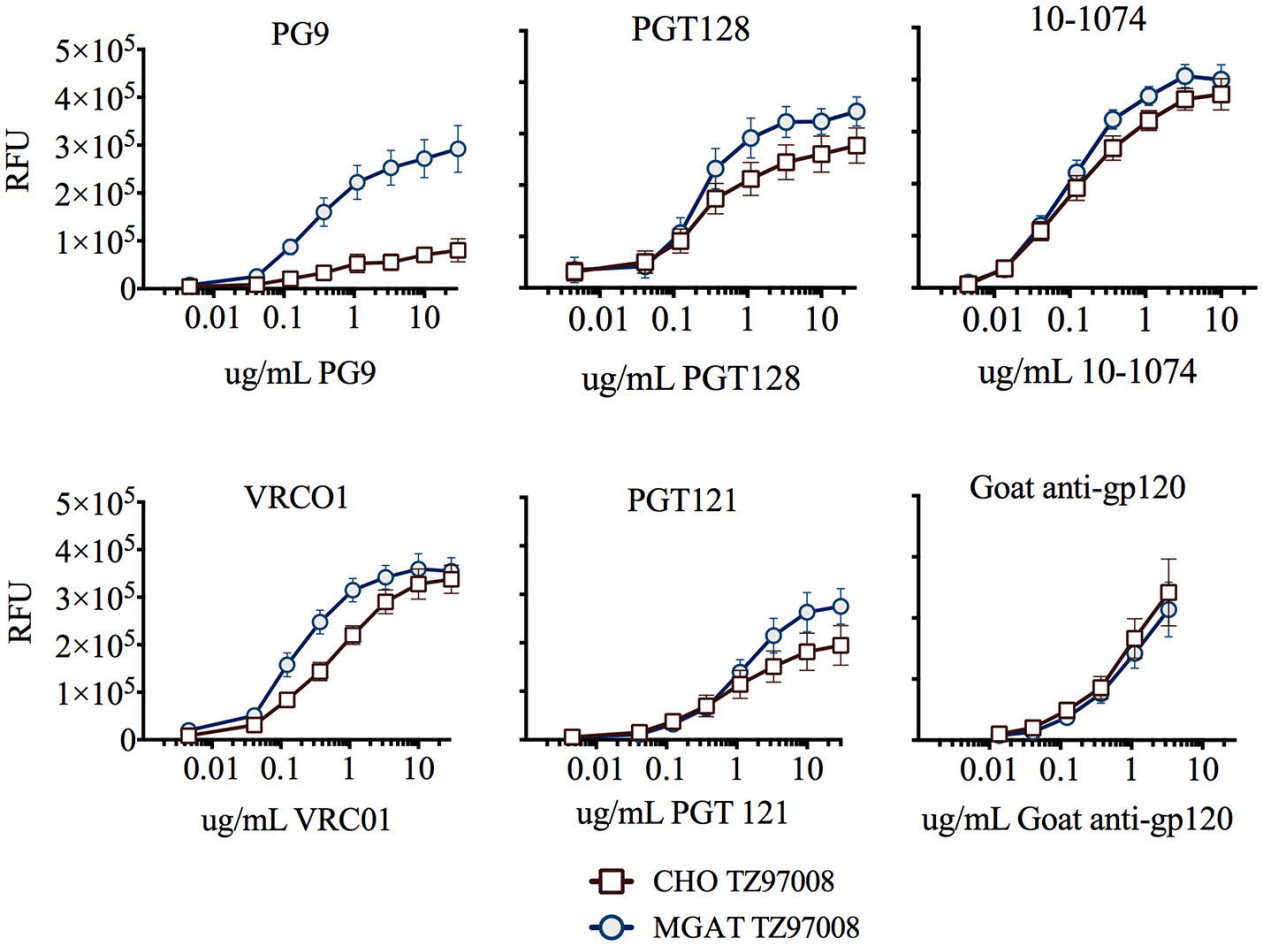
Binding of CHO and MGAT1– CHO produced TZ97008 gp120 to a panel of bNAbs. Purified protein preparations of TZ97008-rgp120 expressed in either CHO (open circles) or MGAT1^−^ CHO cell lines (closed circles) were compared for binding to a panel of bNAbs by FIA. Plates were coated with 2μg/mL of 34.1 specific for the g N-terminal gD tag appended to each protein. The plates were washed, blocked, and incubated with 6μg/mL of purified rgp120. After a subsequent wash, captured antigen was incubated with one of six different bNAbs. The binding of bNAbs to rgp120 was detected with fluorescently labeled goat-anti-human polyclonal antibody. Each curve represents the average of a minimum of triplicate assays.

TZ97008-rgp120 expressed in the MGAT1^−^ CHO or CHO cell lines was assayed for bNAb binding by SPR and fluorescence immunoassay. SPR indicated that production of rgp120 in MGAT1^−^ CHO cells improved TZ97008 binding to the PG9, PGT128, PGT121, and VRC01 bNAbs. The production of TZ97008 rgp120 in the MGAT1^−^ CHO line provided almost four-fold improvement in K_d_ of PGT128 binding, six-fold improvement in K_d_ of PG9 binding, and over ten-fold improvement in K_d_ of VRC01 binding (Table 2). While the VRC01 antibody does not directly contact a glycan, improved binding may be partially explained by better accessibility of protein epitopes in the context of smaller glycoforms. Both the PGT121 and 10-1074 bNAbs bind glycans (based at N332 or N334) at the stem of the V3 domain and derive from the same inferred IgVH germ-line genes (7, 31). The 10-1074 bNAb exhibited binding to both CHO-S and MGAT1^−^ CHO produced rgp120s. However, PGT121 improved binding to CHO derived gp120 as compared to MGAT1^−^ CHO derived rgp120 (Table 2). The binding of bNAbs and a positive control goat polyclonal antibody to the TZ97008 gp120 was also assayed by FIA. The results observed for SPR could be mostly recapitulated for fluorescence immunoassay (Figure 7). There was no apparent difference of either TZ97008 glycoform to the positive control goat polyclonal. However, a small discrepancy was observed in the PGT121 binding, for which FIA indicated improved MGAT1^−^ CHO expressed gp120 binding, while Biacore results showed improved affinity for CHO derived gp120. However, both methods showed that PGT121 is able to bind to TZ97008-rgp120. Overall, the MGAT1^−^ CHO expressed rgp120 displayed better bNAb binding than CHO derived TZ9708 gp120 to three different families of bNAbs, indicating improved antigenic structure of neutralization sensitive epitopes to at least three different regions of the envelope protein.

**Table 2:** Comparative bNAb binding affinities for TZ97008-rgp120 produced in CHO-S or MGAT1– CHO cells.

**bNAb**	Ka*E4 (association)		Kd*E-4 (dissociation)		**Chi2**		Kd*E-9(nM)	
	**MGAT**	**CHO**	**MGAT**	**CHO**	**MGAT**	**CHO**	**MGAT**	**CHO**
PG9	6.51	0.66	11.42	18.95	0.47	0.169	17.56	288
PGT128	6.59	1.92	1.73	3.73	0.71	0.339	2.62	19.44
10-1074	1.6	2.01	6.46	8.68	0.55	0.32	40.51	43.16
VRC01	1.59	0.97	6.64	2.8	0.193	0.041	4.18	28.84
PGT121	0.76	1.15	5.04	3.25	0.16	0.148	65.99	28.22

*Purified rgp120 was assayed for binding to a panel of panel of prototypic bNAbs that target three distinct sites of virus vulnerability of the gp120 envelope protein. These include antibodies to glycan dependent epitopes in the V2 domain glycan (PG9) or the stem of the V3 domain (PGT128, PGT121, and 10-1074), and a glycan independent epitope proximal to the CD4 binding site (VRC01). bNAbs were captured onto a CM5 sensor chip via a covalently immobilized anti-human F_C_ antibody. Serial dilutions of TZ97008 expressed in MGAT1^−^ CHO or CHO cell lines were injected in HBS-EP buffer, at a flow rate of 30 uL/min. Average measurements from blank injection were subtracted from analyte response measurements, and data was rejected if Chi2 values exceeded 1.0, or R_max_ values exceeded 100*.

## Discussion

Here we report: (1) the screening and selection of a clade C gp120 (TZ97008) with improved ability to bind a panel of bNAbs, (2) the development of a stable, high expressing (>1g/L) MGAT1^−^ CHO cell line that limits N-linked glycosylation for improved binding to glycan dependent bNAbs, (3) the development of an improved purification scheme for gp120 produced in the MGAT1^−^ CHO cell line that eliminates the need for immunoaffinity chromatography, and (4) initial biophysical characterization of the purified rgp120 product. Based on screening of a panel of diverse clade C envelope proteins, TZ97008-rgp120 displayed a superior antigenic structure with respect to binding to a panel of prototypic bNAbs. An alignment of the TZ97008 sequence with the LANL clade C consensus sequence indicated that the TZ97008 is similar to the consensus sequence, particularly in relation to the amount and location of N-linked glycosylation motifs. From our screening experiment we found that TZ97008 is one of the few clade C gp120s able to bind the prototypic PG9 and PGT128 bNAbs. Notably, the TZ97008 rgp120 also displayed improved bNAb binding over the TV1 and 1086 clade C strains of which rgp120 immunogens currently being tested in human clinical trials are derived (16).

Previously, rgp120 purification methods required the use of lectin affinity or immunoaffinity columns for purification (23). These resins were required because the high degree of heterogeneity in net charge caused by variation in the sialic acid content precluded the use of conventional ion-exchange purification methods. Indeed, site-specific glycan analysis of gp140 proteins identified up to 70 unique glycoforms occupying a single N-linked glycosylation site (37). Production of HIV vaccine immunogens in the MGAT1^−^ CHO cell line eliminated the need for affinity purification strategies by reducing the net charge and sialic acid heterogeneity of the rgp120 product. The reduced heterogeneity in glycan structure and protein pI, facilitated downstream purification by allowing the use of ion exchange chromatography strategies similar to those described previously (16, 38). The final 3E5 MGAT1^−^ CHO TZ97008 cell line expressed approximately 1200 mg of TZ97008-rgp120 in an 11-day shake flask culture. The two-step chromatography method has a final yield of roughly 50%, resulting in about 600 mg final protein product per L of culture. This yield represents an improvement compared to other cell lines producing gp120 for clinical studies, where yields of 5-100 mg/L have been reported (16, 39). The yields of gp120 described here can be further improved in a setting intended for biopharmaceutical production incorporating regulated fed batch bioreactors, optimized cell growth medium, and improved filtration and purification methods. The recovery process developed for MGAT1^−^ CHO expressed TZ97008-rgp120 was based on its predicted isoelectric point (pI 8.4) and direct IEF measurements. We expect rgp120 from other strains of HIV produced by the MGAT1^−^ CHO cell line to possess similar pIs, and therefore, to also be amenable to purification by this method.

Recently multiple gp120 immunogens have been advanced into human clinical trials (16, 40, 41) in an effort to repeat and verify the results from the RV144 HIV vaccine clinical trials (3). The gp120s employed in these trials, like the gp120s used in the VAX003 (14), VAX004 (15), and RV144 clinical trials (23), were expressed in normal CHO cell lines. Thus, these vaccines failed to incorporate the knowledge gained since the RV144 trials related to the structure of glycans required for the binding of bNAbs. Both FIA and Biacore analysis indicated improved binding for MGAT1^−^ CHO expressed TZ97008 rgp120 as compared to CHO expressed gp120 for the PG9, PGT128, 10-1074, and VRC01 bNAbs. While the improved binding of envelope proteins produced in the MGAT1^−^ CHO cell to bNAbs does not necessarily translate to improved bNAb immunogenicity, the closer emulation of the glycan structures found on infectious virions and required for bNAb binding is a logical first step in the development of an improved vaccine formulation. In principle, improving the immunogenicity of any one of the four major epitopes described in this paper could potentially improve the level of protection achieved from 31% obtained in the RV144 trial to a level of 50% or more thought to be required for regulatory approval (42).

Previous studies comparing HEK 293 expressed TZ97008 have found it to induce comparable cross-clade anti-gp120 titers to a broad diversity of diverse clade C gp120 immunogens (17). Additionally, expression of A244 gp120 in the GNTI^−^ HEK 293 cell line that limits glycoforms to oligomannose terminal glycans did not significantly decrease overall immunogenicity (43). Together, these data suggest that overall immunogenicity of TZ97008 MGAT1^−^ CHO will not significantly change, despite the changes in glycoform and bNAb binding. However, in the face of the historically short lived anti-gp120 response, future studies investigating dosing, different adjuvants, and different immunization schedules will need to be thoroughly performed to understand the full immunogenic potential of these immunogens. How the immunogenicity of MGAT1^−^ CHO TZ97008 rgp120 compares to the original RV144 rgp120 immunogens is an open question, as is the best formulation strategy to enhance antibody responses to glycan dependent epitopes. bNAbs against HIV often contain unusual characteristics such as high levels of somatic hypermutation or long complementarity domain regions that are difficult to elicit in animal models (4, 29, 30, 44, 45). Because of this, the antigenic potential of new vaccine immunogens may only be ultimately determined in human clinical trials. With this perspective in mind, the TZ97008-rgp120 is one a few gp120s that is designed to incorporate glycoforms required for bNAb binding and expressly produced for human clinical trials. The improvements described in this paper can be applied to facilitate the production and clinical testing of other HIV envelope-based vaccine concepts, including gp140 trimers (46) guided immunization (47, 48), DNA prime/gp120 boost protocols (49, 50) vector/gp120s boosts (40, 51) and envelope protein fragments (43, 52).

## Author contributions

RD, SO, and PB contributed to study design. KM contributed to construct design. RD and BY performed binding assays. JR performed alignments, and MW and SO performed cell culture and cell line development. BY and LY designed and performed purification methods. GB provided the MGAT1^−^ CHO cell line and culture system. All authors contributed to manuscript revision.

### Conflict of interest statement

The authors declare that the research was conducted in the absence of any commercial or financial relationships that could be construed as a potential conflict of interest.
